# Regional analysis of UK primary care prescribing and adult service referrals for young people with attention-deficit hyperactivity disorder: from little to very little

**DOI:** 10.1192/bjo.2020.28

**Published:** 2020-04-30

**Authors:** Samuele Cortese

**Affiliations:** Centre for Innovation in Mental Health, School of Psychology, Faculty of Environmental and Life Sciences, University of Southampton, UK; Clinical and Experimental Sciences (CNS and Psychiatry), Faculty of Medicine, University of Southampton, UK; Solent NHS Trust, UK; New York University Child Study Center, USA; and Division of Psychiatry and Applied Psychology, School of Medicine, University of Nottingham, UK

**Keywords:** ADHD, medication, transition

## Abstract

Drawing on data from the Clinical Practice Research Datalink, Price et al reported UK regional variations in primary care prescribing and referral rates to adult mental health services for young people with attention-deficit hyperactivity disorder (ADHD) in transition from child and adolescent mental health services. Overall, considering that around 65% of young adults with childhood ADHD present with impairing ADHD symptoms and up to 90% of individuals with ADHD may benefit from ADHD medications, the study by Price et al shows that the rate of appropriate treatment for youngsters in the transition period varies from low to very low across the UK. As such, there is a continuous need for education and training for patients, their families, mental health professionals and commissioners, to eradicate the misconception that, in the majority of the cases, ADHD remits during adolescence and to support the devolvement of appropriate services for the evidence-based management of adult ADHD across the UK.

## ADHD in transition

In past years, I have attended several working groups meetings aimed at fostering evidence-based practices in the management of attention-deficit hyperactivity disorder (ADHD) in the UK. One of the key issues that is often discussed in these type of meetings is around the challenges related to the continuity of care during and beyond the transition from child and adolescent mental health services (CAMHS) to adult mental health services (AMHS), which should occur between the ages of 16 to 18. Arguably, this is a very vulnerable phase in life for a number of youngsters, including those with ADHD, because of pressures to define career pathways and living arrangements, that may increase the levels of stress and require additional coping skills and resilience.

It is important to appreciate any geographical disparity in the management of ADHD to better tailor any specific action aimed at improving the quality of care. Therefore, ‘What do data tell us about regional variations in the prescriptions of ADHD medications during the transition period ?’ is a frequent question I have been asked at these meetings. Like the rest of my colleagues attending the working groups, I remember feeling slightly uncomfortable and somehow embarrassed myself by the paucity of data that I was aware of, having to admit ‘Well, ehm… I am not sure there are peer-reviewed, published data on this’.

## The study by Price et al

Fortunately, Price and colleagues^1^ have contributed to fill this gap. Drawing on the Clinical Practice Research Datalink (CPRD), a large primary care database containing records of about 11 million patients, the aims of the study by Price et al^1^ were to: (a) examine regional differences in the prevalence of prescriptions of ADHD medications in primary care for young people aged 16–19 years in the UK; (b) estimate the proportion of patients referred to AMHS; (c) map regional variations in prescribing patterns and referrals rates to the location of services for adult ADHD.

The study by Price et al^1^ should be considered within the context of the current knowledge on the persistence of ADHD after adolescence, as well as of the available guidelines and evidence synthesis on ADHD treatment. An often cited meta-analysis^2^ showed that up to 65% of young adults with a childhood diagnosis of ADHD present with impairing ADHD symptoms and up to 71% with functional impairment, even without a formal diagnosis of ADHD. For school-age children with ADHD, the 2018 NICE guidelines^3^ recommend the use of medication (in case of persistence of significant impairment in at least one domain of life after initial group-based focused support), with the following hierarchy: (a) methylphenidate; (b) lisdexamfetamine (or dexamphetamine if lisdexamfetamine is not well tolerated); (c) atomoxetine or guanfacine. Behavioural parent training is recommended only when there is a comorbid oppositional defiant disorder or conduct disorder. For adolescents, cognitive–behavioural therapy is suggested if impairing symptoms are still present after the pharmacological treatment. In adults, medication is the recommended first-line approach, with methylphenidate or lisdexamfetamine (or dexamphetamine if lisdexamfetamine not well tolerated) as first line, followed by atomoxetine. Non-pharmacological treatments are indicated in case of difficulty adhering to medication, if medication is ineffective or not well tolerated, or if patients prefer them.

Overall, these recommendations are in line with available meta-analytic evidence. As for short-term effects, the most comprehensive network meta-analysis^4^ of randomised controlled trials (RCTs) (time points closest to 12 weeks) showed superiority of many ADHD medications over placebo in reducing the severity of ADHD core symptoms, with effect sizes for amphetamines (from ~0.8 to 1) that are not only the highest for the medications used in psychiatry, but also among the highest in general medicine.^5^ Furthermore, evidence from observational within-individual designs to account for confounding by indication shows a protective effect of ADHD medications on a number of important outcomes in children (for example unintentional physical injuries) and in adults (for example criminal acts).^6^

As for the long-term effects of medications, although as a result of ethical and practical aspects it is challenging to prove them using the standard RCT design, a number of discontinuation RCTs (i.e. RCTs in which patients who have been on medication are either randomised to continue the treatment or to placebo) are emerging, showing the persistence of effects beyond the ‘short term’.^7^

The percentage of individuals with ADHD who should be treated with medication is not well established, but sequential RCTs show that only 18–23% of patients might respond to an initial trial of behavioural interventions.^8^ It has also been reported that around 90% of patients with ADHD respond to at least one type of medication for ADHD.^9^ Finally, based on rigorous meta-analytic evidence, the effects of non-pharmacological treatments on core symptoms of ADHD in children and adolescents are uncertain, when relying on masked/probably masked ratings,^10–13^ even though additional methodological sound research in adults is needed.

Given this evidence, one should expect a substantial persistence of pharmacological treatment in young people with a history of childhood ADHD. But this is not what the study by Price et al^1^ tells us. In the area with the highest prescribing rate (Scotland), the percentage of patients with at least one prescription of ADHD medication was 47% at 14–15 years and 27% at 19–20 years; in the region with the lowest rates of prescription (Yorkshire & Humber), the rate dropped from to 27% to 6%. Furthermore, the mean age of termination (or interruption) of pharmacological treatment was 16.6 years (s.d. = 2.63), in sharp contrast with the notion that in the majority of cases, impairing ADHD symptoms persist after adolescence.

It should be highlighted that these figures provide only an underestimated rate of individuals with ADHD who are properly managed with the pharmacological treatment. Indeed, one of the key lessons that the field has learned from the well-known Multimodal Treatment of ADHD (MTA)^14^ study and from efforts to implement the MTA approach in the real clinical world^15^ is that, to be effective, ADHD medications need to be optimised with an accurate titration and follow-up. As such, ‘at least one prescription’ is just a rough indication of effective treatment.

Additionally, given the limitations of real-world databases such as the CPRD, Price et al^1^ could not assess adherence to treatment. Therefore, the first finding of the study can be summarised as follow: there is a regional variability in the prescription of ADHD medication across geographic areas in the UK, but overall the rate of prescriptions (and, importantly, of appropriate treatment) varies from low to very low. This finding should be considered in the context of the documented underrecognition of ADHD, which varies by deprivation^16^ and the gap between the recognised prevalence of ADHD versus its community prevalence in the UK.^17^

In relation to the second study objective, the authors found that the average percentage of patients referred to AMHS was 11% only, ranging from 4% to 21%, and the figures were even lower when excluding individuals with a prescription of any other psychotropic medications (average: 7%, from 3% to 11%). Finally, Price et al^1^ found no clear identifiable associations between reduction in prescribing, referral rates to AMHS and location of services specialised in the care of adults with ADHD. However, this finding should be considered with caution, as CPRD data referred to the period 2005–2013, while the service mapping was completed in 2018.

The study findings may be accounted for by a number of possible factors. First, it is interesting to note that the mean age of termination of medication coincides with the period when young people complete the General Certificate of Secondary Education and may leave school, suggesting that they may deem that treatment for a condition that they erroneously perceive as only an ‘academic’ problem is not necessary anymore. Second, it is possible that general practitioners (GPs) do not continue the prescription because of a lack of shared care plans agreements, paucity of ADHD specialised services or, in some areas (for example Nottinghamshire^18^) guidelines preventing GPs from prescribing ADHD medications in adults. It is also possible that a number of prescriptions continue being renewed without proper specialist input, which would be a major problem *per se*. Finally, ADHD medications might not be prescribed because these young people access non-conventional medications thought to improve ADHD or non-pharmacological approaches; in both cases, this would suggest that a sizeable portion of patients are not treated as per current guidelines.

## Implications of the study by Price et al

Regardless of the possible explanation, the study by Price et al^1^ suggests that there is a clear need for education on the management of ADHD during the transition period. Until 20–30 years ago, ADHD was thought to remit in adolescence, and indeed the 2000 NICE guidelines^15^ recommended that treatment with ADHD should normally be stopped in adolescence. But the field has come a long way since. With a large body of evidence currently available (2955 hits on 2 February 2020 with a simple PubMed search: adult* [ti] AND (ADHD [ti] or attention-deficit [ti])) we do know now that ADHD can have serious consequences in adults as well. There is a need to provide education and training to patients, families, GPs and other professionals involved in the care of ADHD, as well as commissioners. In particular, GPs and adult mental health professionals should receive education and training on the importance of monitoring medication use and supporting adherence.

Public awareness campaigns addressed at adolescents with ADHD should be promoted, to strengthen adherence to treatment in adulthood. The role of professional groups such as the UK Adult ADHD Network (https://www.ukaan.org/) is pivotal in this regard. Additional research is needed on the psychosocial impact of ADHD, the efficacy, effectiveness, and safety of pharmacological interventions for adults with ADHD, and the profile of young adults with ADHD that require transition to adult services, with the goal to develop local protocols to monitor and support the mental health of young people with ADHD.

From my side, I will keep on providing my modest contribution delivering training and attending working groups, where I am sure I will feel less embarrassed next time I am asked about regional variations in prescribing of ADHD medications for young people in transition from CAMHS to AMHS.
